# Shrimp (*Penaeus monodon*) preservation by using chitosan and tea polyphenol coating combined with high‐pressure processing

**DOI:** 10.1002/fsn3.2939

**Published:** 2022-05-28

**Authors:** Lihang Chen, Dexin Jiao, Bihe Zhou, Chen Zhu, Jingsheng Liu, Dali Zhang, Huimin Liu

**Affiliations:** ^1^ College of Food Science and Engineering Jilin Agricultural University Changchun China; ^2^ National Engineering Laboratory for Wheat and Corn Deep Processing Changchun China

**Keywords:** coating treatment, high‐pressure processing, shelf life, shrimp

## Abstract

The present work investigated the effects of high‐pressure processing (200 and 400 MPa, 5 min) combined with chitosan‐tea polyphenol (1.5% and 0.5% [w/v], respectively) coating to improve the quality and stability of shrimp (*Penaeus monodon*) during 28 days of storage. The chemical (pH, TVB‐N, TBARS), microbiological, textural, chromatic characteristics, protein oxidation, and endogenous enzyme activities of shrimps were regularly evaluated. Results showed that the combination treatment exerted a better intense antimicrobial effect, stabilized shrimp's freshness, and resulted in lower pH and TVB‐N than the control sample. Also, combined treated samples had better oxidative stability than a single treatment until the end of shelf life. Although combination treatment had no significant effect on endogenous proteases, the combined use of 400 MPa high‐pressure and chitosan‐tea polyphenol coating was most effective in inhibiting the bacteria and improved the hardness and chromatic characteristics of shrimp within the storage.

## Graphical abstract

400 MPa high‐pressure and chitosan‐tea polyphenol coating was most effective in inhibiting the bacteria and improving the hardness and color of shrimp within the storage.

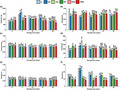

## 1 INTRODUCTION

Shrimp is among the most popular international traded fishery products for its desirable flavor and nutritional value (FAO, 2020). Shrimp is an excellent source of protein, with a protein content of about 70% by dry weight. In addition, shrimp meat is low in fat and is a typical high‐protein, low‐fat food (Bindu et al., 2013). Nonetheless, several biochemical and physical modifications caused by endogenous enzymes and microorganisms directly influence shrimp's nutritional and sensory quality during storage. Frozen storage is the most common way to extend the shelf life of shrimp products. However, protein denaturation caused by freezing and thawing would directly affect the quality, such as texture properties, water holding capacity, and flavor (Zhang et al., 2018).

High‐pressure processing has been practically used to preserve foods such as juices, cured meats, and aquatic products. It can inhibit microorganisms and prolong the shelf life of cold chain foods to offer numerous opportunities for developing novel food products (Ginson et al., 2015). Therefore, all procedures such as pressure and holding time should be considered when designing an optimal high‐pressure processing for safe and good sensory quality foods. However, high‐pressure processing also has some undesirable effects. The main changes occurred at more than 200 MPa leading to an increase in pH and whiteness, a decrease in water holding capacity, and initiation of lipids oxidation (de Oliveira et al., 2017).

Chemical preservatives such as sodium metabisulfite are currently used to maintain the quality of shrimp (Galvão et al., 2017). There is growing interest in studying active materials with antibacterial and antioxidant capacities considering food safety and shelf life extension. Edible coatings, such as chitosan and whey protein, are widely being explored to preserve fishery products, efficiently preventing moisture loss, color deterioration, lipid oxidation, inhibiting enzyme activities and enhancing shelf‐life (Umaraw et al., 2020; Yu et al., 2019). Chitosan biofilms reduce the formation of reactive oxygen and effectively prevent the colonization of microbiota communities by forming a cross‐linked film on the food surface (Hussain et al., 2021). Natural preservatives have been broadly used in the food industry, among which tea polyphenols are the most widely used (Alishahi & Aïder, 2012). For example, tea polyphenols can inhibit polyphenol oxidase activity and slow down the melanosis of shrimp (Sae‐Leaw et al., 2017; Shiekh et al., 2019). Besides, the combination treatment of high‐pressure processing and chitosan‐based edible films can effectively inhibit the growth of microorganisms (Albertos et al., 2015; Gómez‐Estaca et al., 2018).

Through the fence effect, the combination of multiple processing methods may be more conducive to extending the shelf life of product. Many studies have focused on the edible coating combined with other food processing technologies such as plasma (Wong et al., 2020), modified atmosphere packaging (Xiong et al., 2020), and irradiation (Zhang et al., 2017) to enhance the antibacterial and antioxidant properties of food. Summarizing the published studies, it may have a complementary effect between high‐pressure processing and chitosan‐tea polyphenol coating.

Therefore, this study aimed to evaluate the effects of high‐pressure processing combined with chitosan‐tea polyphenols coating on the quality and stability of shrimp (*Penaeus monodon*) during storage. Shrimps were divided into six groups and stored for 28 days. Meanwhile, pH, TVB‐N, TVC, TBARS, and chromatic parameters of samples were determined. The protein oxidation was determined by the disulfide bond and carbonyl content. At the same time, the texture properties of shrimp and the activities of cathepsin B, H, L, and calpain in their proteins were determined.

## 2 MATERIALS AND METHODS

### 2.1 Preparation of coating solution

Chitosan powder (deacetylation degree: approximately 95%; viscosity: 200‐400 mPa.s) was purchased from Acmec Biochemical Co., and green tea polyphenols were purchased from Yuanye Bio‐Technology Co. Constituent compounds of tea polyphenols used in this study are listed in Table S1. Coating solution was composed of 1.5% chitosan (m/v), 1% acetic acid (v/v) and 0.5% green tea polyphenols (m/v), homogenizing (T18, I.K.A.) at 10,000  *g* for 10 min. The microemulsions were degassed by ultrasound (KQ3200DB; Ultrasonic Instruments Co., Ltd, Kunshan, Jiangsu, China) for 10 min and then used immediately for coating. The whole operation is completed in an asepsis room.

### 2.2 Preparation of samples

Fresh shrimps (Penaeus monodon) with an average length of 10 cm and weight of 10 g were purchased in December 2020 from a local market in Changchun, Jilin, China, and transported to the laboratory in iced condition. Shrimps were immediately washed in 4°C sterile distilled water, headed manually, and drained for 5 min.

### 2.3 Combination treatment

The six treatments were defined as follows. The sample without any treatment was used as a control (C). Single‐treated samples by high pressure (200 MPa and 400 MPa for 5 min) and chitosan‐tea polyphenol coating treatment without high‐pressure treatment were noted as C2, C4, and TP, respectively. The coating samples followed by high pressure (200 MPa and 400 MPa for 5 min) were noted as TP2 and TP4. Shrimps were immersed in solutions with a shrimp/solution ratio of nearly 1:5 (w/v) and slightly shaken for 5 min. Shrimps were placed in a strainer and then drained for 5 min at 4°C to remove the excess coating. The headless shrimps were vacuum‐packed in EVOH multilayer films for high‐pressure treatment. The entire operation was performed on ice. Pressure treatment was carried out in an high‐pressure machine (600 MPa/30 L, Bao Tou KeFa High Pressure Technology Co., Tianjin, China). Shrimps were subjected to the pressurization conditions of the corresponding batches, 200 and 400 MPa pressure with a holding time of 5 min. The other parameters of high‐pressure processing are shown in Table S1. Both treated and untreated samples were stored in insulated boxes with a shrimps/ice ratio of 1:2 (w/w) and kept in a refrigerator at 4°C ± 1°C for 28 days storage. Molten ice was removed and replaced every 2 days.

### 2.4 Chromatic characteristics of shrimp meat

The chromatic parameters of shrimps were measured using a spectrophotometer (CM 5, Konica Minolta, Singapore) in terms of the universally accepted CIELAB color scale. The second section of the abdomen muscle of shrimps was selected for the determination. Chromatic characteristics were expressed in L*, a*, and b*. The W* (whiteness) was computed as, W* = 100 – [(100 − L*)^2^ + a*^2^ + b*]^0.5^.

### 2.5 Textural profile analysis and shear force

The Textural profile analysis (TPA) of shrimps was performed using a TA. XT Plus texture analyzer (Stable Micro Systems, Haslemere, Surrey, UK). The smooth abdomen muscle (the second ventral segments) of shrimps was selected for the TPA. The test parameters were set as follows: cylindrical probe P5, test speed 1 mm/s, both pretest speed and post‐test speed are 2 mm/s, and compressed depth 50%. The smooth abdomen muscle (perpendicular to the muscle fibers axis of the third ventral segments) of shrimps was selected for the shearforce test. The test parameters were set as follows: Warner‐Bratzler blade, crosshead speed 0.5 mm·sec^−1^, initial height 15 mm, and the blade runs 13 mm.

### 2.6 Chemical and microbiological analysis

The TVB‐N content was determined according to Chen, Jiao, Yu, et al. (2022). The results were expressed in mg N/100 g of sample. Based on the Chinese National Standard (GB2733‐2015), the limit of TVB‐N values was set as 30 mg N/100 g for shrimp.

A sample of 1 g was homogenized with 9 ml of water distilled CO_2_ free at 12,000 rpm for 30 s. The pH value was measured using a pH meter (MP220, Mettler Toledo) at 20°C ± 2°C.

The TBARS of shrimps was performed as described by (John et al., 2005). The results were expressed as mg MDA/kg of sample.

The total aerobic viable count of shrimps was determined. Samples (10 g) were mashed and transferred to a sterile bag (Bkmam®), and then diluted 10 times sterile water and homogenized (BagMixer 400CC, Inter Science) for 2 min. Appropriate serial dilutions of homogenates (0.1 ml each) were spread on sterile agar plates. After pouring the media, the plates were cultured for 72 h at 30°C and the bacterial counts were enumerated.

### 2.7 Extraction of myofibrillar protein

The shrimp muscle was used for myofibrillar protein extraction according to the method described by Chen, Jiao, Liu, et al. (2022). Breifly, shrimp meat was mixed with pre‐chilled phosphate buffer (pH 7.0) at a ratio of 1:9. The mixture was homogenized (T18, I.K.A.) for 30 s at 12,000 × g, then centrifuged (Allegra X‐30R, Beckman Coulter) at 2000 × g for 15 min at 4°C. The precipitate was then homogenized in four volumes of salt solution (0.1 M NaCl), centrifuged (2000 × g for 15 min), and washed three times. The final pellet was collected as MP and suspended in phosphate buffer (pH 7.0). The protein concentration of the prepared myofibrillar protein was measured using the Biuret method (Gornall et al., 1949).

### 2.8 Disulfide bond

The disulfide bond was determined according to Chen, Jiao, Yu, et al. (2022). Briefly, 100 μl 4 mg/mL myofibrillary protein solution was mixed with 1 ml buffer (8 M urea, 3 mM EDTA, 1% SDS, 0.1 M Na2SO3, 1% NTSB, 0.2 M Tris–HCl [pH 9.5]). After mixing, the reaction was avoided light at 37°C for 25 min. The absorbance at 412 nm was measured with the microplate analyzer (FLUOstar Omega, BMG LABTECH). The results were expressed in nmol/mg protein of simple:

(1) $$M\;\left(\frac{\text{nmol}}{\mathrm{mg}}\text{protein}\;\right)=\frac{\Delta A_{412}}{b\times E\times C}\times \frac{1.1\;\mathrm{mL}}{0.1\;\mathrm{mL}}\times 10^{6}\;$$

Type: Δ A412 under 412 nm absorbance minus the contrast absorbance;

b: The light transmission distance at the dosage of 200 μl per well of the enzymatic plate analyzer, that is 0.588 cm;

E: The molar extinction coefficient (m^−1^ cm^−1^), the disulfide bond is 13,600;

C: protein concentration (mg/mL).

### 2.9 Carbonyl content

The determination of carbonyl content was referred to Colombo et al. (2016) with slight modification. Briefly, two aliquots of 0.5 ml of 8 mg/ml myofibrillar protein were reacted with 2 ml 2,4‐dinitrophenylhydrazine (DNPH) solution (10 mM, dissolved with 2 M HCl), 2 ml 2 M HCl solution as a blank. Samples were vortexed for 15 s every 25 min in the dark at room temperature for 1 h. The reaction was terminated by adding 2.5 ml 20% TCA and centrifuged at 11,000 × *g* for 3 min. The residue was washed three times with 2 ml ethyl acetate/absolute ethanol (1:1, v/v) and then collected. 6 ml of 6 M guanidine hydrochloride was added to dissolve the residue and then centrifuged at 11000 × g for 3 min. The absorbance of the supernatant was measured at 370 nm. The carbonyl content (nmol/mg protein) was calculated using the molecular absorption coefficient of 22,000 M^−1^ cm^−1^.

### 2.10 Specific proteolytic activities determination

The enzymatic extract was prepared following the previously described methodology (Yang et al., 2015). The fluorescence of AMC (7‐Amino‐4‐methylcoumarin) was measured by Lumina Fluorescence Spectrometer (Thermo). The test parameters were set: excitation wavelength 360 nm; emission wavelength 460 nm; slit width 10 nm slits. The activities of cathepsin B, H, L, and calpain were determined by using the fluorogenic substrates (ONTORES Co. Zhejiang, China) Z‐Arg‐Arg‐AMC, L‐Arg‐AMC, Z‐Phe‐Arg‐AMC, and N‐succinyl‐Leu‐Tyr‐AMC, respectively. The enzyme activities were expressed as U/mg protein/min. The protein concentration of the enzyme extracts was determined by the BCA Protein Quantitation Kit (Beyotime Biotechnology).

### 2.11 Statistical analysis

All experiments were performed at least three replicates. Mean values and standard deviation were calculated using R (3.6.3) for the individual samples. One‐way analysis of variance (ANOVA) was performed using Benjamini and Hochberg adjustment. The significant differences were defined as *p* < .05.

## 3 RESULTS AND DISCUSSION

### 3.1 pH and TVB‐N values

**FIGURE 1:**
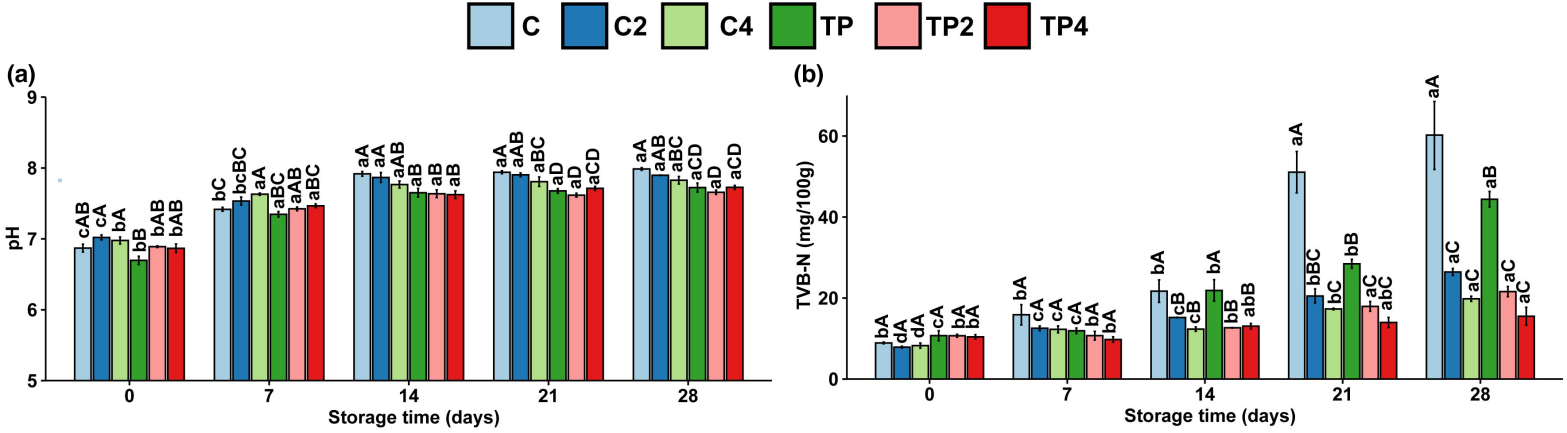
Changes in (a) pH and (b) total volatile base nitrogen of samples with different treatments during iced storage. Values are expressed as mean ± standard deviation; lower case letters (a–c) mean in the same pressure, different times are significantly different, and superscript capital letters (A–C) mean in the same time, different pressures are significantly different (*p* < .05)

Aquatic products will still undergo physicochemical changes during refrigeration, and pH is often used to evaluate the freshness of aquatic products since microbial or enzymatic activities modify it. As shown in Figure 1A, the initial pH value of fresh shrimp was 6.87 and reached 8.19 at the 28‐day of storage. The initial pH value was markedly lower than the values reported for *Gracilaria gracilis* (7.20) (Balti et al., 2020). Species, feeding conditions, harvest period, and degree of stress before processing could contribute to this pH difference. The pH increased after pressure treatment on the first day of storage, which could be due to the changes in the structures of protein exposed to alkaline amino acid residues (de Oliveira et al., 2017).

Shamshad et al. (1990) mentioned that the pH value increased with storage time and confirmed a positive correlation between pH and overall acceptability. Three quality grades can be distinguished based on pH value: good quality (<7.7), poor but acceptable quality (7.70–7.95), and unacceptable quality (>7.95) (Marshall & Wiese‐Lehigh, 1997). A pH value of 7.95 ± 0.04 was obtained for untreated shrimp after 21 days of storage, and it could be considered that the quality of the shrimp was unacceptable. Moreover, the pH of samples treated with high pressure and coating did not exceed 7.7 within 28 days. In particular, using 200 MPa high pressure combined with chitosan‐tea polyphenol coating minimized the increased pH values of samples. The synergistic effect of coating and high pressure significantly impacted the shrimp freshness compared to high pressure alone. The rising tendency was relatively inhibited by chitosan‐tea polyphenol coating, which may be because both chitosan and tea polyphenol could retard microbial growth and prevent protein hydrolysis.

The TVB‐N level, related to the enzymatic degradation of amino acids, is still considered a valuable index of fresh and lightly preserved seafood. As shown in Figure 1B, the initial TVB‐N content of fresh shrimp was 8.9 mg/100 g, which was close to the value previously reported for *Gracilaria gracilis* (8.3 mg N/100 g) (Balti et al., 2020). TVB‐N values in all shrimp samples increased with storage time. A significantly higher TVB‐N value was obtained in untreated shrimp. The range of untreated shrimp exceeded 30 mg/100 g (upon the limit is usually regarded as spoiled) before 21 days of storage, while the shrimp treated at 200 and 400 MPa were no more than 30 mg/100 g during the 28 days of storage. The current study showed that coatings combined with high pressure reduced TVB‐N levels during storage. The above results agreed with previous studies that the TVB‐N values of salmon carpaccio treated with high pressure and chitosan coating were decreased (Gómez‐Estaca et al., 2018).

### 3.2 TVC changes

**FIGURE 2:**
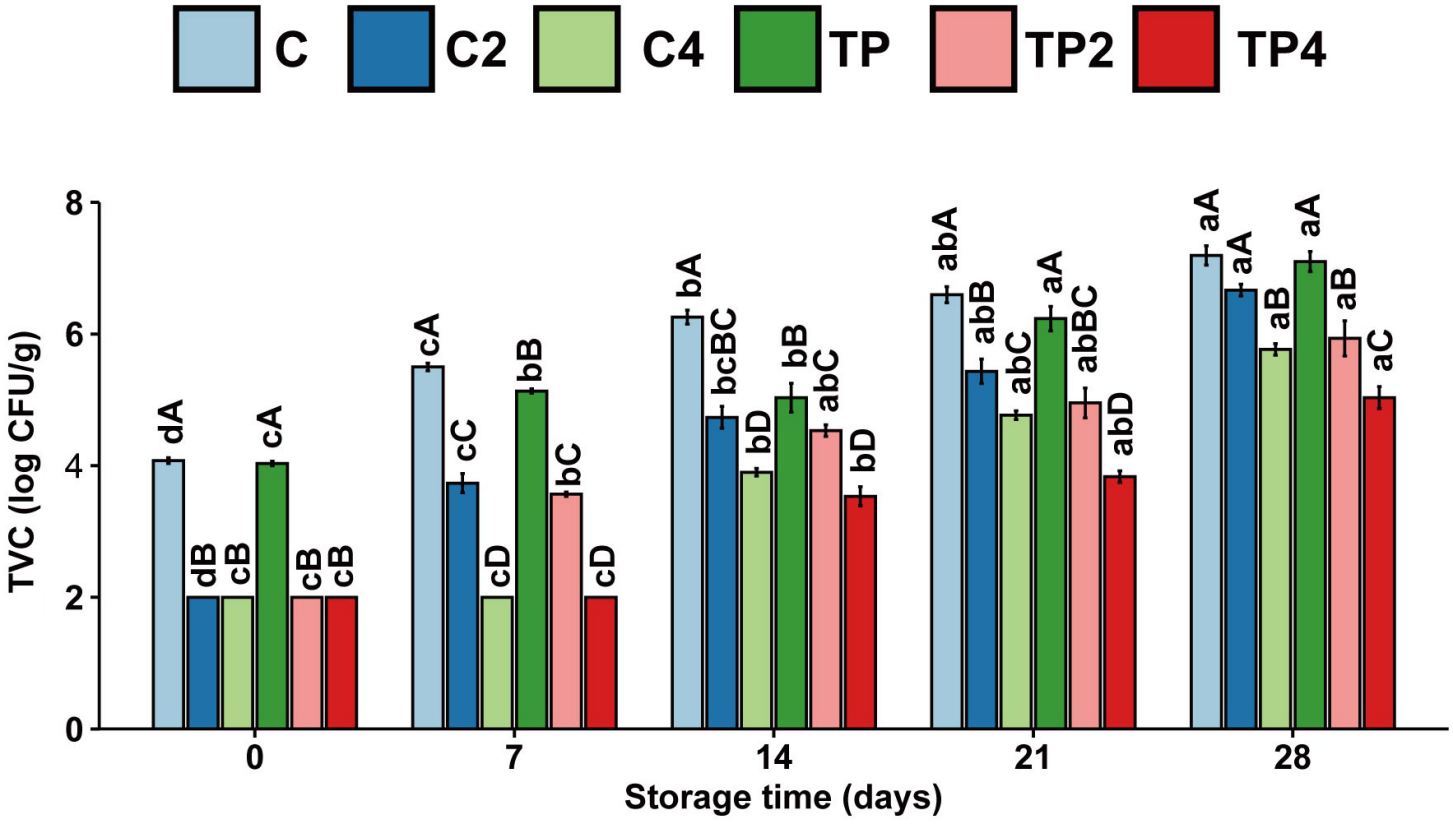
Changes in total viable bacteria (TVC) of samples with different treatments during iced storage. Values are expressed as mean ± standard deviation; lower case letters (a–c) mean in the same pressure, different times are significantly different, and superscript capital letters (A–C) mean in the same time, different pressures are significantly different (*p* < .05)

Seafood products often contain many bacteria due to their growing environment, which is a risk to the health of consumers, resulting in premature deterioration of the product or even food poisoning. According to the International Commission on Microbiological Specifications for Foods, 7 log10 CFU/g of TVC level limit is recommended (Balti et al., 2020). As shown in Figure 2, the initial TVC was around 4.1 log CFU/g, which was markedly higher than that observed by Balti et al. (2020). This difference may be correlated with the fishing and handling of the product. The TVC levels of the pressure‐treated samples were significantly reduced on the first day of storage, which was attributed to the excellent ability of the pressure treatment to improve the quality of crustacean microorganisms (Ginson et al., 2015). The increase in TVC level was highest in the untreated shrimps for all the sampling days and from the 28 days of storage; this value (7.2 ± 0.3 log_10_ CFU/g) slightly exceeds the damage limit. In the present work, both high‐pressure processing and coating treatment inhibited the accumulation of microorganisms during storage. In addition, the combined treatment had a better bacteriostatic effect. The TVC level in TP4 sample was 5.0 log_10_ CFU/g on the last day of storage. High‐pressure processing can delay the growth of Enterobacteriaceae, mesophilic bacteria, psychrotrophic bacteria, proteolytic bacteria, Pseudomonas spp., H2S producing bacteria, lactic acid bacteria, yeast, and mold in shrimp (Ginson et al., 2015). Chitosan exhibited a bacteriostatic effect on Gram‐negative bacteria, Enterobacteriaceae, and Vibrio cholerae (Benhabiles et al., 2012). Meanwhile, chitosan was soluble in 1% acetic acid. Thus, the chitosan/pH synergetic effect is probably one explanation of the antimicrobial effect (Alishahi & Aïder, 2012).

### 3.3 Chromatic parameters analysis

**FIGURE 3:**
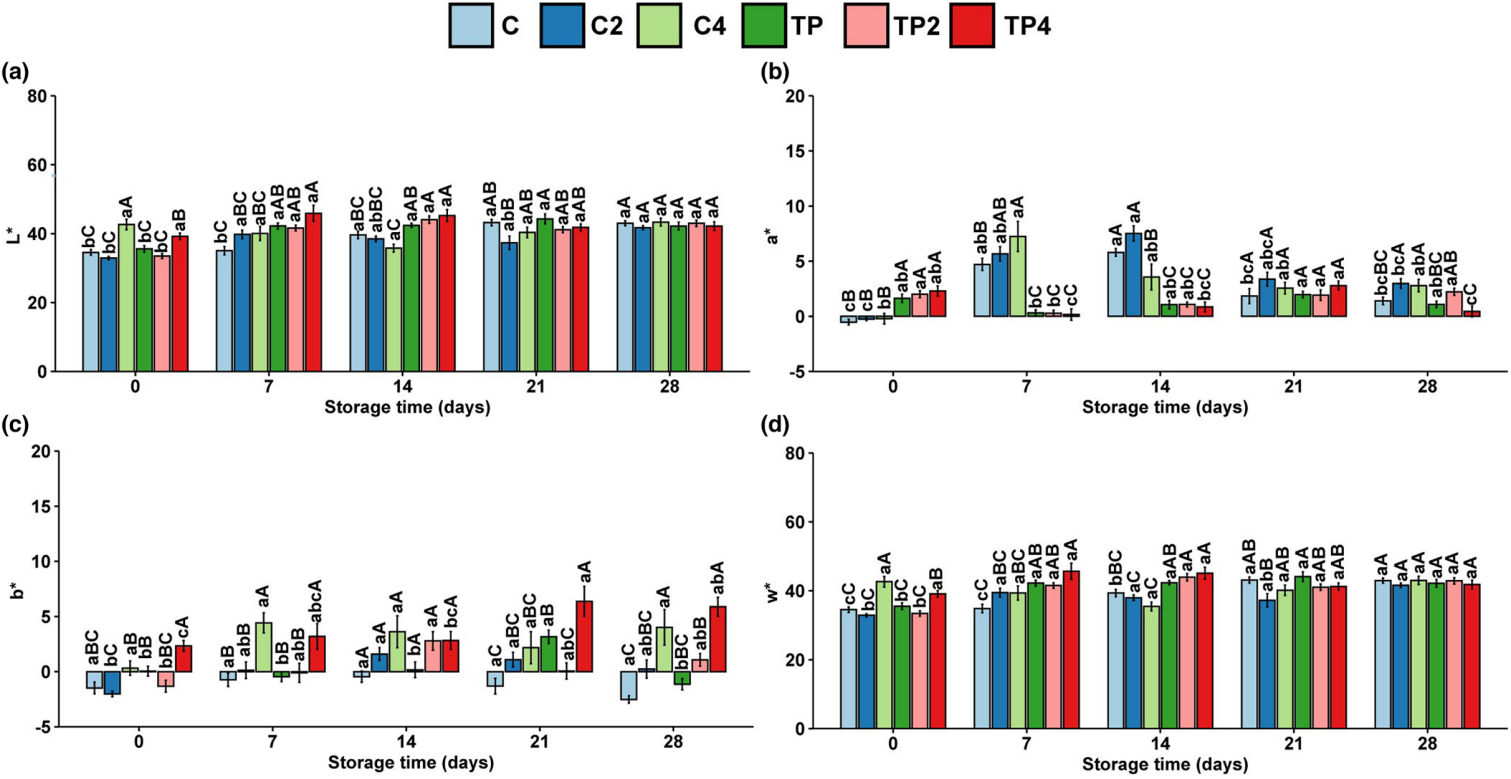
Changes in chromatic parameters of samples with different treatments during iced storage. Results of (a) L*, (b) a*, (c) b*, (d) W*. Values are expressed as mean ± standard deviation; lower case letters (a–c) mean in the same pressure, different times are significantly different, and superscript capital letters (A–C) mean in the same time, different pressures are significantly different (*p* < .05)

For food preference, it is believed that “the first taste is almost always with the eye” (Hutchings, 1999). The effect of high‐pressure processing and chitosan‐tea polyphenol coating on chromatic parameters of shrimp during storage is shown in Figure 3. The L* value is usually related to the protein status of the muscle. A significant increase in L* was observed in 400 MPa high‐pressure‐treated groups, while this phenomenon was not significant during storage because pressure‐induced protein denaturation is relatively stable during chilled storage. a* variation of treated shrimp showed several fluctuations during storage. The a* value of untreated samples increased from −0.53 (day 0) to 5.8 (day 14). These values were significantly (*p* < .05) different from coating‐treated samples. As shown in Figure 3C, the blueness variation of 400 MPa pressure‐treated shrimp increased during storage. During handling, processing, and storage, polyphenol oxidases (PPO) in the shrimp body quickly react and cause melanosis. Blackening is initially present in the carapace of the cephalothorax and then spreads to the exoskeleton of the abdominal area, primarily in the area where the cuticles are connected (Sae‐Leaw & Benjakul, 2019a). Tea polyphenols with PPO inhibitory activity effectively retard melanosis of crustaceans during the storage (Sae‐Leaw & Benjakul, 2019b). Previous studies highlighted that the +Δa* (red bias) in shrimp appearance co‐occurs with the formation of melanosis (Balti et al., 2020) and chitosan coatings could improve the color quality of shrimp (Wang et al., 2018). Our study confirmed that chitosan‐tea polyphenol coatings could avoid the development of red (+Δa*) color in shrimp.

### 3.4 Changes in TBARS, disulfide bonds, and carbonyl content

**FIGURE 4:**
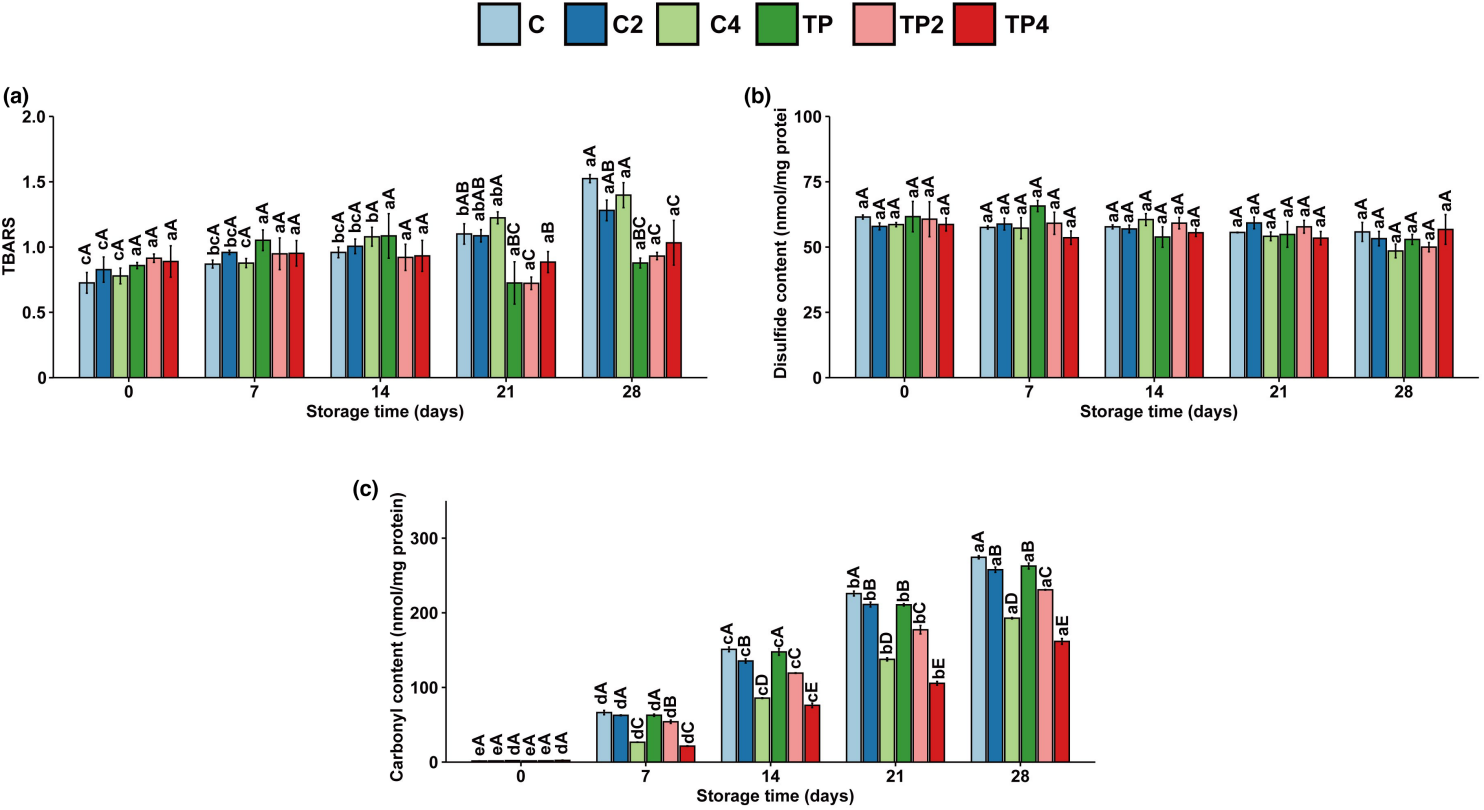
Changes in (a) thiobarbituric acid reactive substances, (b) disulfide content, and (c) carbonyls of samples with different treatments during iced storage. Values are expressed as mean ± standard deviation; lower case letters (a–c) mean in the same pressure, different times are significantly different, and superscript capital letters (A–C) mean in the same time, different pressures are significantly different (*p* < .05)

The effect of HPP on lipid oxidation of aquatic products is mainly evaluated by quantitative analysis of secondary oxidation products such as thiobarbituric acid reactants (TBARS). As shown in Figure 4, the initial TBARS value of shrimp meat was 0.73 mg MDA/kg. The rise in TBARS value might be related to unsaturated fatty acids oxidation and the partial dehydration of shrimp. Several studies have reported that high pressure may induce oxidation (de Oliveira et al., 2017). A significant increase in TBARS values was observed in pressure‐treated salmon, cod, and mackerel (Rode & Hovda, 2016) and squid (Zhang et al., 2016). However, in this study, high‐pressure treatment has no significant effect on lipid oxidation (except on day 21, the value of 400 MPa treated shrimps was higher). The TBARS increased in untreated and single pressure‐treated samples during storage (*p* < .05), while the value of coating samples was relatively stable. Lipid oxidation could be inhibited by coating treatment containing chitosan and tea polyphenols. This observation was similar to the refrigerated red drum fillets treated with grape seed extract and tea polyphenols (Li et al., 2013).

Thiol groups from cysteine residues are prone to attacks by reactive oxygen species, resulting in disulfide cross‐linking. Covalent bonds are pressure insensitive at least at commercial pressures below 600 MPa; therefore, the high pressure used in this study had no significant effect on disulfide bonds (Mozhaev et al., 1996). The disulfide bond in the shrimps was 45–70 nmol/mg of myofibrillar protein (Figure 4B). The content of disulfide bonds in each treatment group did not change significantly during refrigeration, indicating that the protein cysteine residues in shrimp muscle were not oxidized.

The formation of protein carbonyls is one of the most useful general indicators for assessing oxidation. As shown in Figure 4C, there was a remarkable increase in the protein carbonyl content with storage time. The initial carbonyl content of fresh shrimp was 1.5 ± 0.1 nmol/mg protein and reached 274.4 ± 3.6 nmol/mg protein at the 28‐day of storage. In the present work, both high‐pressure processing and coating treatment inhibited the accumulation of carbonyls during storage. Polyphenols have strong free radical scavenging ability and antioxidant activity because of their phenolic hydroxyl groups (Xie et al., 2019). The lipid oxidation is higher at the surface than at the inner part of the meat (Bolumar et al., 2021). The coating delays oxygen exchange between the shrimp and the outside world, slowing oxidation.

### 3.5 Textural properties analysis

**FIGURE 5:**
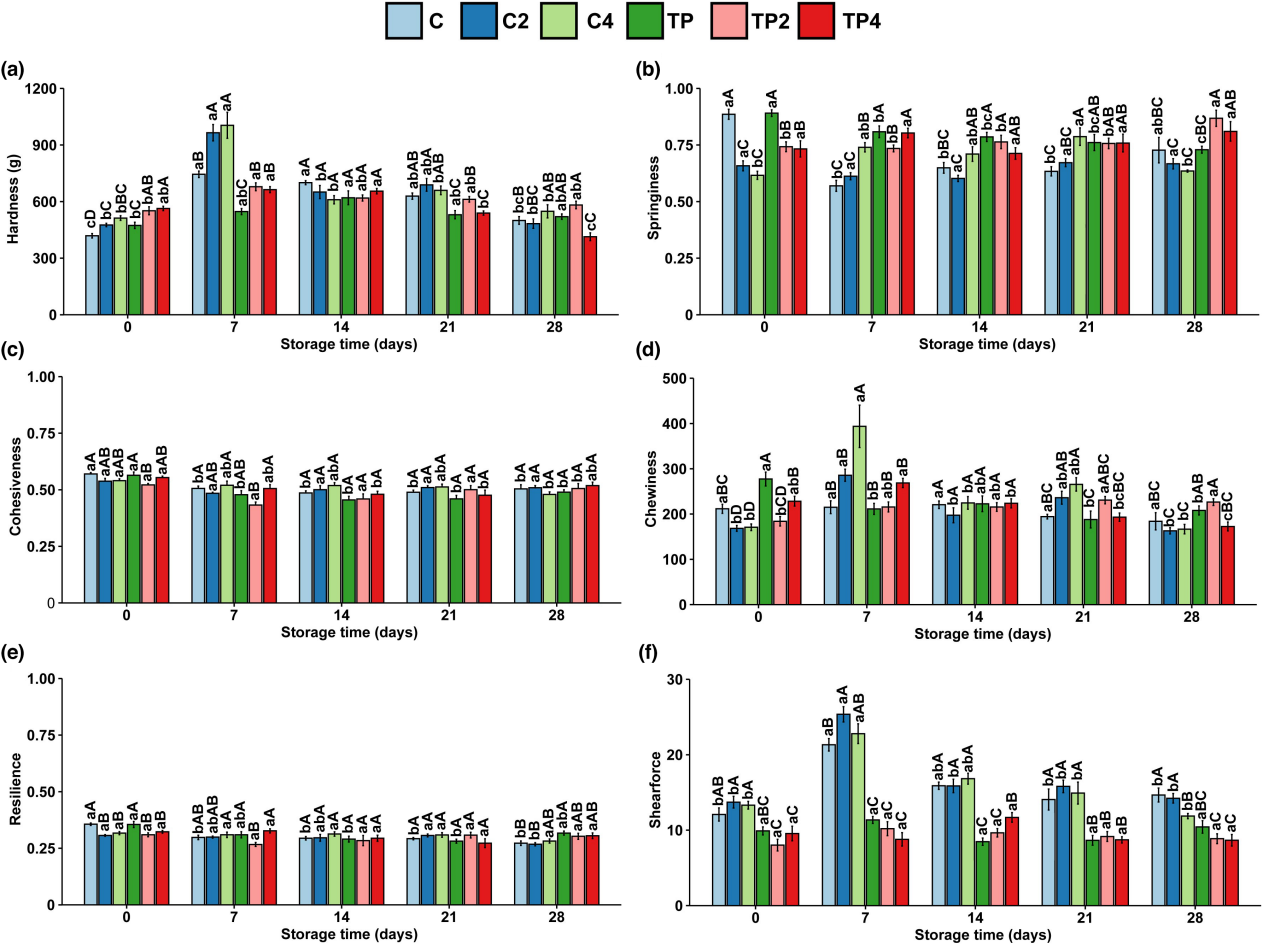
Changes in textural properties of samples with different treatments during iced storage. Results of (a) hardness, (b) springiness, (c) cohesiveness, (d) chewiness, (e) resilience, and (f) shearforce. Values are expressed as mean ± standard deviation; lower case letters (a–c) mean in the same pressure, different times are significantly different, and superscript capital letters (A–C) mean in the same time, different pressures are significantly different (*p* < .05)

Figure 5 shows texture parameters in different treatments of shrimps. Compared to the untreated sample, the initial hardness of samples gradually increased with the elevated pressure; in contrast, the springiness was decreased (Figure 5A‐B). On the other hand, other textural parameters such as chewiness and shearforce differ significantly between untreated and coating treated shrimps during the storage (Figure 5). Different groups had different initial values of mechanical properties before refrigeration, which was mainly caused by pressure‐induced protein denaturation (Chen, Jiao, Liu, et al., 2022).

Texture parameters varied most from day 0 to day 7 throughout the storage period. The hardness of shrimps increased by 178%, 203%, 196%, 99%, 143%, and 118%, respectively. The active coating based on chitosan and tea polyphenol appears to impact the texture of refrigerated shrimp. The PCA plot evaluated differences in texture properties under different treatments (Figure S1). The first two principal components explained 71.1% of the total variation (44.4 and 26.7% for PC1 and PC2, respectively). To further evaluate the effect of various treatments on the texture properties of shrimp. The initial position of each sample was taken as the starting point, and sum of the distance between each time point and the starting point during the period in the score scatter plot, it was 23.0 (C), 11.8 (C2), 12.4 (C4), 18.1 (TP), 6.9 (TP2), and 8.4 (TP4), respectively. In comparison, shrimps treated with a combination of pressure and coating reduced variability in texture properties during the 28‐day storage period. The pressure within 400 MPa had no significant effect on the protease activity in shrimp meat. Nonetheless, the mechanical properties of shrimp treated at 400 MPa were stable during chilled storage, probably because pressure‐induced protein denaturation did not change over time (Chen, Jiao, Liu, et al., 2022). (Wang et al., 2018) also found that chitosan coating effectively delayed the texture deterioration of shrimp during storage.

### 3.6 Proteolytic activities

It is generally accepted that proteases in muscle and viscera, or proteases secreted by microorganisms, are the main detrimental factors in proteolysis and deterioration of muscle texture during refrigerated storage. Cathepsin and calpain are the most widely studied proteases (Kemp et al., 2010).

**FIGURE 6:**
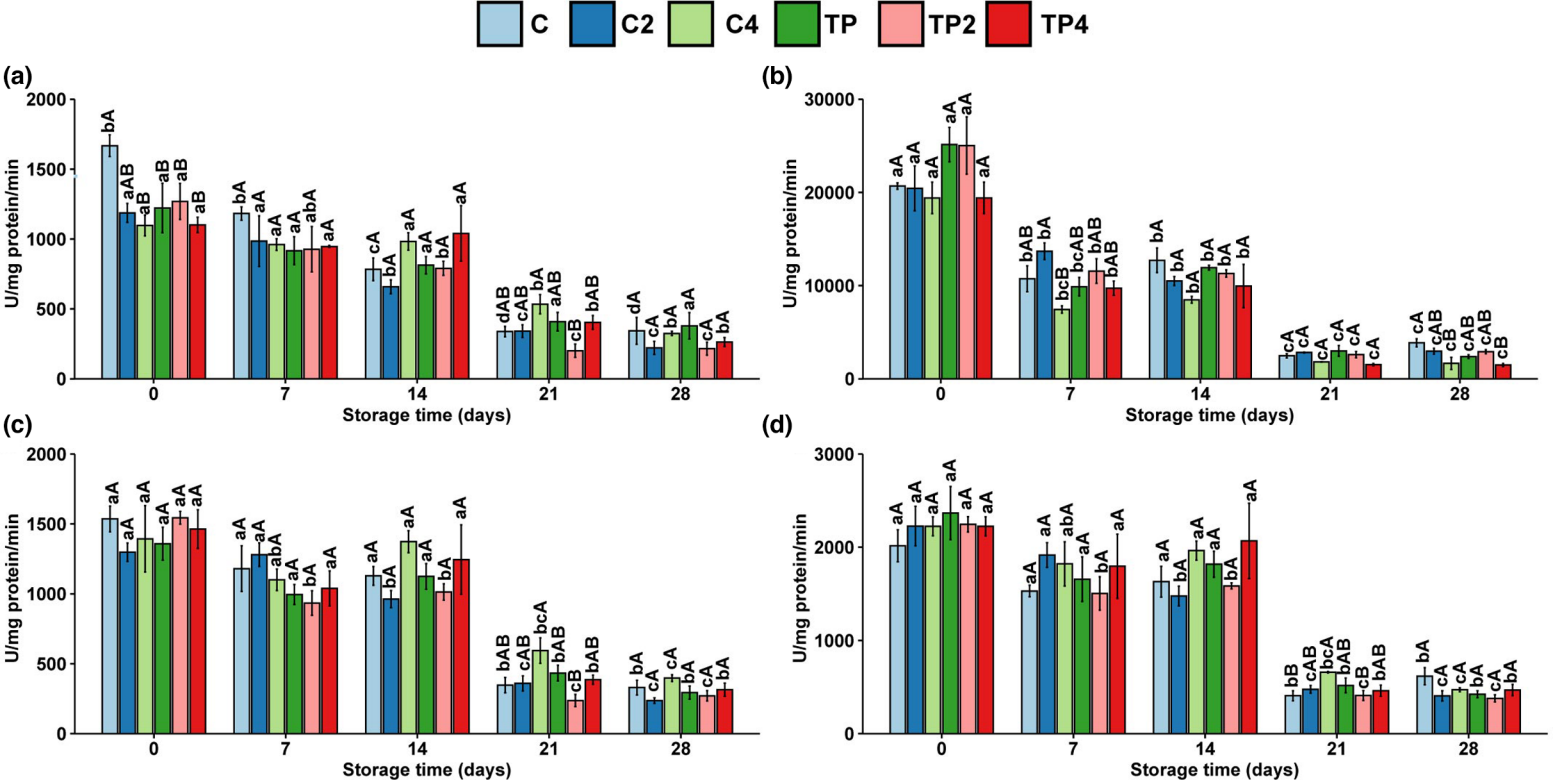
Changes in enzymatic activities of samples with different treatments during iced storage. The activity of (a) cathepsin B, (b) cathepsin H, (c) cathepsin L, and (d) calpains. Values are expressed as mean ± standard deviation; lower case letters (a–c) mean in the same pressure, different times are significantly different, and superscript capital letters (A–C) mean in the same time, different pressures are significantly different (*p* < .05)

As shown in Figure 6, high‐pressure processing has different inactivation effects on different enzymes. Cathepsin B was relatively sensitive to high pressure compared with others. The activities of cathepsin B immediately decreased by 24–34% after high pressure, but during refrigeration, there was no significant difference between untreated and pressure‐treated samples. This result was similar to that reported by Yu, Yan, et al. (2018), cathepsins were only affected at higher pressures (>400 MPa), and this difference gradually diminished with prolonged refrigeration. The enzymatic activity of all enzymes decreased to 15–35% of the initial enzymatic activity after 28‐day refrigeration. The activity of all enzymes gradually decreased during remained at 15–35% of the initial activity by day 28. Although previous research reported that chitosan‐based coatings improved the quality of refrigerated fish fillets by alleviating endogenous enzyme‐induced proteolysis (Feng et al., 2016; Yu, Regenstein, et al., 2018). Neither high pressure nor coating treatment significantly affected endogenous enzymes in this study. The coating treatment was beneficial in inhibiting the oxidation of lipids and proteins but had no significant effect on proteases, which may be because the former mainly occurs on the surface of the muscle. In contrast, the latter occurs inside the muscle.

## 4 CONCLUSIONS

In this study, the effects of high‐pressure processing combined with chitosan‐tea polyphenol coating on the quality of shrimp (*Penaeus monodon*) during chilled storage have been comprehensively analyzed. The combination treatment inhibited lipid peroxidation and the bacterial proliferation of shrimp stored in the refrigerator. It further retained shrimp's original sensory qualities, such as color and texture properties, efficiently lowed pH and TVB‐N increase and prolonged freshness. Thus, high‐pressure processing (400 MPa, 5 min) combined with chitosan‐tea polyphenol (1.5% and 0.5% (w/v), respectively) makes it possible to extend the shelf life and obtain stable and safe processed products.

## CONFLICT OF INTEREST

The authors declare that they have no known competing financial interests or personal relationships that could have appeared to influence the work reported in this paper.

## ETHICAL APPROVAL

Ethics approval was not required for this research.

## Supporting information


Appendix S1
