# Supplementary material for: Shrimp (Penaeus monodon) preservation by using chitosan and tea polyphenol coating combined with high‐pressure processing
Source: Food Sci Nutr. 2022 May 28;10(10):3395–404. doi: 10.1002/fsn3.2939 (PMC9548362; doi:10.1002/fsn3.2939)
Supplement: Supplementary file 1 — Appendix S1 [file FSN3-10-3395-s001.docx]

**Shrimp (*Penaeus monodon*) preservation by using chitosan** **and tea polyphenol coating combined with high pressure processing**

Lihang Chen ^1,2^, Dexin Jiao ^1,2^, Bihe Zhou ^1,2^, Chen Zhu ^1,2^, Jingsheng Liu ^1,2^, Dali Zhang ^1,2, *^, and Huimin Liu ^1,2,**^

^1^ College of Food Science and Engineering, Jilin Agricultural University, Changchun, Jilin 130118, China; liuhuimin@jlau.edu.cn

^2^ National Engineering Laboratory for Wheat and Corn Deep Processing, Changchun, Jilin 130118, China;  [liuhuimin@jlau.edu.cn](mailto:%20liuhuimin@jlau.edu.cn)

^*^ Correspondence: [spzdl@126.com](mailto:spzdl@126.com)

^**^ Correspondence: liuhuimin@jlau.edu.cn

**Supplementary File**

**Table S1.** Constituent compounds of tea polyphenols^*^

| **Compounds** | **Content (mg/g)** |
| --- | --- |
| Epigallocatechin gallate (EGCG) | 43.04 ± 0.22 % |
| Epicatechin gallate (ECG) | 18.42 ± 0.12 % |
| Epigallocatechin (EGC) | 10.32 ± 0.37 % |
| Epicatechin (EC) | 6.65 ± 0.23 |
| Gallic acid (GA) | 1.21 ± 0.01 |

*: Data from Xie, X., Liu, X., Fei, Y., Zhao, Q., & Jin, S. (2020). Single and combined effects of tea polyphenols and edible chitosan coating on the shelf life improvement of refrigerated dagger-tooth pike conger (Muraenesox cinereus).

**Table S2.** The parameters of ultra-high pressure

| **Pressure**  **（MPa）** | **Come-up Time (s)** | **Holding Time (s)** | **Decompression Time (s)** | **Max Pressure During Holding (MPa)** | **Min Pressure During Holding (MPa)** | **Temperature**  **(℃)** |
| --- | --- | --- | --- | --- | --- | --- |
| 200 | 50 | 300 | 2 | 208 | 195 | 14-15 |
| 400 | 90 | 300 | 3 | 412 | 388 | 14-15 |


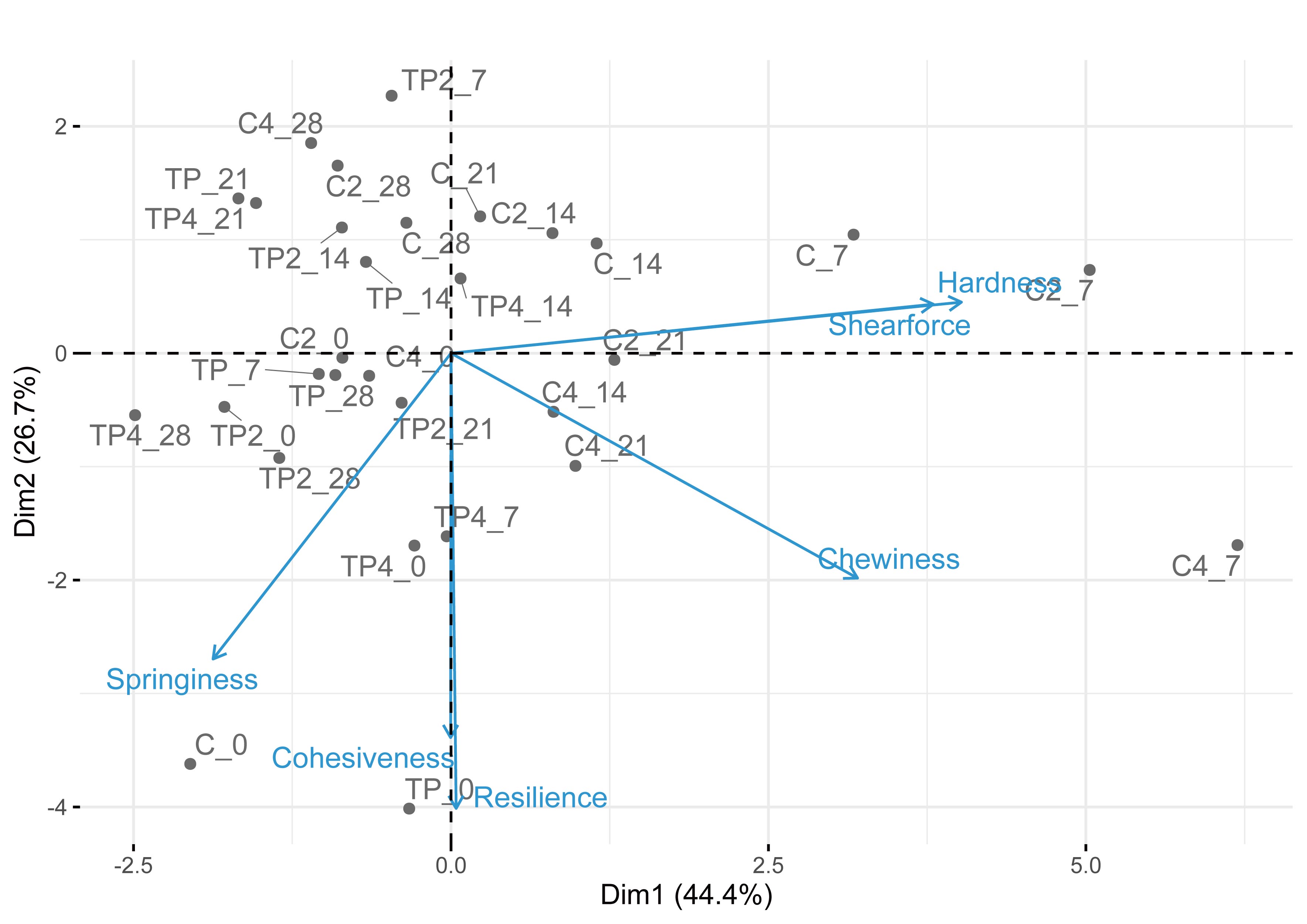


**Fig. S1** Principal component analysis of the texture properties change in each group during iced storage.
